# Selective inhibition of c-Myb DNA-binding by RNA polymers

**DOI:** 10.1186/1471-2091-5-15

**Published:** 2004-11-04

**Authors:** Oddmund Nordgård, Tor Ø Andersen, Odd S Gabrielsen

**Affiliations:** 1 Department of Molecular Biosciences, University of Oslo, P.O.Box 1041 Blindern, N-0316 Oslo, Norway; 2 Department of Haematology and Oncology, Rogaland Central Hospital, P.O.Box 8100, N-4068 Stavanger, Norway

## Abstract

**Background:**

The transcription factor c-Myb is expressed in hematopoietic progenitor cells and other rapidly proliferating tissues, regulating genes important for proliferation, differentiation and survival. The DNA-binding domain (DBD) of c-Myb contains three tandemly arranged imperfect repeats, designated Myb domain R_1_, R_2 _and R_3_. The three-dimensional structure of the DBD shows that only the second and third Myb domains are directly involved in sequence-specific DNA-binding, while the R_1 _repeat does not contact DNA and only marginally affects DNA-binding properties. No structural information is available on the N-terminal 30 residues. Since deletion of the N-terminal region including R_1 _plays an important role in oncogenic activation of c-Myb, we asked whether this region confers properties beyond DNA-binding to the neighbouring c-Myb DBD.

**Results:**

Analysis of a putative RNA-binding function of c-Myb DBD revealed that poly(G) preferentially inhibited c-Myb DNA-binding. A strong sequence-selectivity was observed when different RNA polymers were compared. Most interesting, the poly(G) sensitivity was significantly larger for a protein containing the N-terminus and the R_1_-repeat than for the minimal DNA-binding domain.

**Conclusion:**

Preferential inhibition of c-Myb DNA binding by poly(G) RNA suggests that c-Myb is able to interact with RNA in a sequence-selective manner. While R_2 _and R_3_, but not R_1_, are necessary for DNA-binding, R_1 _seems to have a distinct role in enhancing the RNA-sensitivity of c-Myb.

## Background

The transcription factor c-Myb is regulating genes involved in proliferation and differentiation during hematopoiesis in vertebrates (reviewed in [1,2]). c-Myb is expressed at high levels in hematopoietic progenitor cells, but becomes down-regulated when the cells reach terminal differentiation. The critical role of c-Myb in the development of hematopoietic cells is emphasized by the embryonic lethality observed in mice with a *c-myb*^*null *^mutation, caused by failure of fetal hepatic hematopoiesis [3]. c-Myb expression has also been detected in other rapidly proliferating tissues such as hair follicles and immature epithelial cells from colon, respiratory tract, skin and retina [4,5]. c-Myb is essential for early T cell development [6] and several c-Myb target genes play an important role during T cell development, like CD4, TCRγ, TCRδ and RAG-2 [1,7]. The best characterized c-Myb target gene is chicken *mim-1*, which is encoding a secretable component of granules found in normal promyelocytes [8,9].

The Myb family of proteins is defined by the presence of a well-conserved DNA-binding domain (DBD) composed of Myb repeats [10]. Each repeat consists of about 50 amino acids with three regularly spaced tryptophans forming a hydrophobic core [11-13]. The c-Myb DBD contains three tandem imperfect Myb repeats (R_1_, R_2 _and R_3_), located in the N-terminus of the protein. Determination of the structure of the c-Myb DBD has revealed that each repeat folds into three well-defined helices forming a helix-turn-helix-related structural motif, where the last helix in R_2 _and R_3 _makes specific DNA contacts (recognition helices) [13-15]. The c-Myb protein harbours two functional domains in addition to the DBD: a central activation domain and a C-terminal negative regulatory domain. The viral counterpart of the chicken c-*myb *gene, v-*myb*, found in the AMV and E26 viruses, has deletions in both ends, leading to a v-Myb protein lacking the N-terminus, most of the first Myb repeat (R_1_) and a large part of the C-terminal negative regulatory domain (reviewed in [16]).

The c-Myb DBD binds specifically to the sequence PyAAC(T/G)G, termed the Myb recognition element (MRE) [17-19]. The minimal sequence-specific DBD consists of the two carboxy-terminal Myb repeats, R_2_R_3 _[20,21]. The role of the first Myb repeat, R_1_, is not fully understood. It has been shown to be dispensable for specific DNA-binding [20,22], but bears striking similarities to the other repeats regarding sequence and structure [23]. Some groups have reported that R_1 _stabilizes the protein-DNA complex [24,25] and it has been proposed to allow for more flexibility in the downstream region of the Myb recognition sequence [26]. According to the recent three-dimensional structure of the c-Myb DBD, the R_1 _repeat does not contact DNA directly [15]. However, a long-distance electrostatic interaction is suggested to stabilize the protein-DNA complex. Its free position in the complex makes it possible that it could be involved in other functions as well. This possibility is supported by the fact that *v-myb*-like truncation of the N-terminus of c-Myb (until the end of R_1_) has been shown to be sufficient for oncogenic transformation of chicken bone marrow cells [27]. The R_1 _repeat could serve as a special regulatory module, either acting as a target for molecular interactions or being subject to post-translational modifications.

In the present work we address whether the N-terminal region including the R_1 _repeat confers properties beyond DNA-binding to c-Myb DBD. We sought evidence for a putative RNA-binding function, analogous with several other transcription factors harbouring dual DNA-RNA binding properties. The motivation for investigating RNA-interaction was the design of the c-Myb DBD, built of repeating modules, a design resembling the structural logic of zinc finger proteins. Some well-studied zinc fingers have been found to use subsets of the repeats as RNA- or DNA-binding units. The classical example is the *Xenopus *zinc finger protein TFIIIA, which acts as a DNA-binding activator of 5S ribosomal RNA genes [28,29]. TFIIIAalso forms a stable complex with 5S rRNA in *Xenopus *oocytes [30,31]. The nine zinc fingers of TFIIIA contribute differentially to DNA- and RNA-binding; the N-terminal triplet (1–3) dominating the DNA recognition event while the middle triplet (4–6) is more important for RNA-binding [32,33]. Another example of a zinc finger with similar dual properties is the Wilms tumor suppressor gene 1 (*WT1*) [34-36]. One splice variant (the +KTS isoform) seems to be a better RNA-interacting form that co-localizes with splicing proteins in nuclear speckles, whereas another variant (the -KTS isoform) interacts stronger with DNA and co-localizes with transcription factors [34,35].

The capability of specific interaction with both DNA and RNA is not restricted to the zinc finger family of proteins. The homeodomain protein bicoid of *Drosophila *acts both as a transcription factor, activating zygotic segmentation genes during blastoderm formation, and as a regulator of mRNA translation by binding to the mRNA of another homeodomain transcription factor, caudal [37,38]. Interestingly, the homeodomain bears structural similarities to the Myb domain, especially with respect to the presence of a helix-turn-helix-related motif [12,13,15,21]. A final example is p53, which has been reported to bind to both single-stranded DNA and RNA in addition to its established role as a sequence-specific DNA-binding protein (reviewed in [39]). Murine p53 and human Cdk4 translation have actually been shown to be regulated by p53 mRNA binding.

Based on these occurrences of dual nucleic acid interactions, we asked whether a similar design was found in c-Myb. In particular, we raised the question whether the N-terminal region including R_1 _might be implicated in RNA-binding rather than DNA-binding. As a test of our proposed RNA-binding function of c-Myb, we investigated the effect of different homoribopolymers on c-Myb DNA-binding. The homoribopolymer polyguanylic acid (poly(G)) strongly inhibited the sequence-specific DNA-binding of c-Myb, while poly(A), poly(C) and poly(U) did not, indicative of an RNA-binding activity. The same phenomenon, although weaker, was observed for A- and B-Myb. An order-of-addition experiment indicated that poly(G) bound directly to c-Myb in competition with DNA. Interestingly, the DBD construct containing the N-terminus and R_1 _was significantly more sensitive to poly(G) than the minimal DBD. Thus, the N-terminus including R_1 _seems to be important for the RNA-sensitivity of c-Myb DBD.

## Results

**Figure 1:**
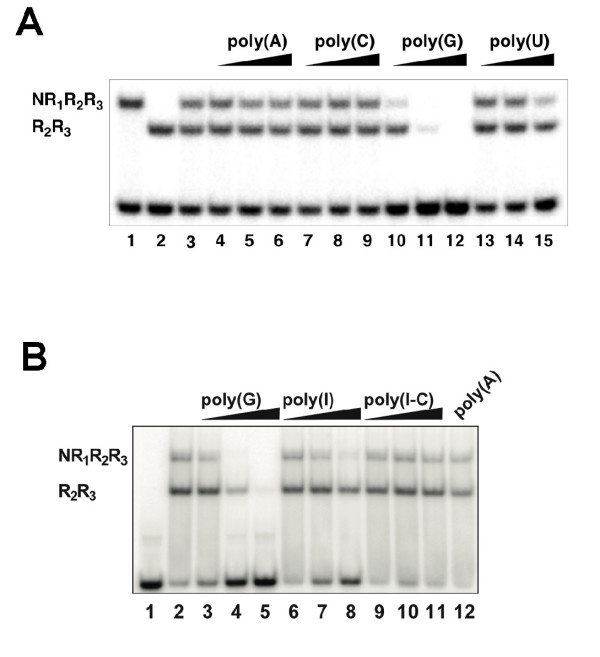
Inhibition of c-Myb DNA-binding by homoribopolymers. Panel A: NR_1_R_2_R_3 _(40 fmol) and R_2_R_3 _(20 fmol) in mixture were incubated with 30 fmol MRE-containing DNA probe and 1 ng (lane 4,7,10 and 13), 10 ng (lane 5, 8, 11 and 14) and 100 ng (lane 6, 9, 12 and 15) of homoribopolymers poly(A) (lane 4–6), poly(C) (lane 7–9), poly(G) (lane 10–12) and poly(U) (lane 13–15). The binding reactions were incubated for 15 minutes at 25°C and subsequently analyzed by EMSA and phosphorimaging. Lane 1, 2 and 3 show binding reactions without homoribopolymer addition, with NR_1_R_2_R_3 _alone in lane 1, R_2_R_3 _in lane 2 and the mixture of both in lane 3. Panel B: NR_1_R_2_R_3 _and R_2_R_3 _were combined in a binding reaction as in panel A, but now in the presence of 1 ng, 10 ng or 100 ng of the ribopolymers poly(G) (lanes 3–5), poly(I) (lanes 6–8), poly(I-C) (lanes 9–11) and 100 ng poly(A) (lane 12), respectively. Lane 1 shows free probe, whereas lane 2 shows binding reaction without ribopolymer addition.

**Figure 2:**
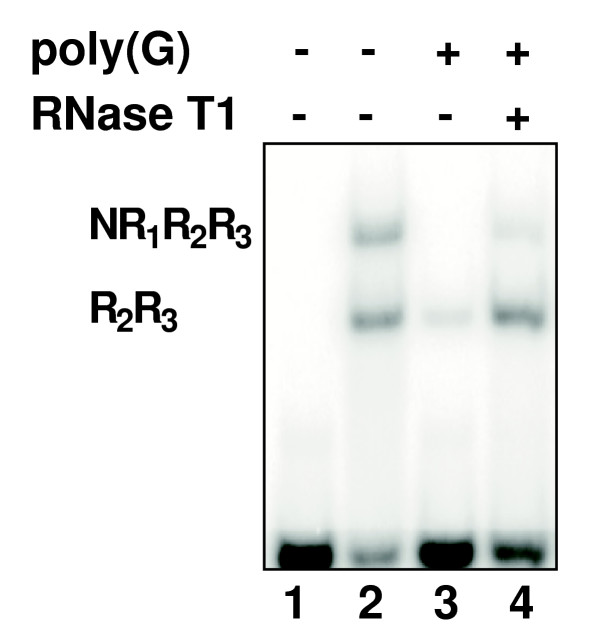
RNase treatment relieves poly(G)-mediated inhibition of c-Myb DNA-binding. NR_1_R_2_R_3 _and R_2_R_3 _were incubated with the MRE-containing DNA probe in the presence of 20 ng poly(G) homoribopolymer (lanes 3 and 4). The sample shown in lane 4 was in addition incubated with 2000 U RNase T1. To allow for RNase T1 mediated degradation of poly(G), all samples were incubated for 30 minutes at 37°C prior to addition of Myb-protein mixture. Binding reactions were subsequently incubated for 15 minutes at 25°C and analyzed by EMSA and phosphorimaging. Lanes 1 and 2 show free probe and binding reaction without homoribopolymer addition, respectively.

Differential binding to homoribopolymers has been used as evidence for RNA-binding activity of various proteins [40-45]. To investigate whether c-Myb and its R_1 _repeat is involved in RNA-binding we first examined the effects of homoribopolymers on c-Myb DNA-binding. The sequence-specific DNA-binding was analyzed by the electrophoretic mobility shift assay (EMSA) using two purified recombinant human c-Myb protein domains, NR_1_R_2_R_3 _(amino acid 1–192) and R_2_R_3 _(amino acid 89–192), in mixture. The assay was performed in the presence of increasing amounts of the homoribopolymers poly(A), poly(C), poly(G) and poly(U) (Fig. 1A). The DNA-binding of both proteins was strongly inhibited by poly(G) but not by the other polymers. NR_1_R_2_R_3 _was most sensitive, being inhibited when as little as 1 ng poly(G) was added. We also tested two artificial RNAs, poly(I*C) and poly(I). The former is a duplex RNA, and the latter is a variant of poly(G) less prone to forming unusual structures, but retaining some of the pairing properties of guanosines. The duplex did not affect DNA-binding, while poly(I) caused some inhibition at high concentrations (Fig. 1B). To confirm that the observed inhibition by poly(G) was indeed due to the added RNA, we carried out the poly(G) inhibition experiment in the presence and absence of RNase T1, an endoribonuclease that specifically cuts RNA at the 3'-end of guanosine residues. As shown in Fig. 2, DNA-binding was no longer inhibited by poly(G) after RNase T1 treatment, confirming the RNA-dependence of the inhibition.

**Figure 3:**
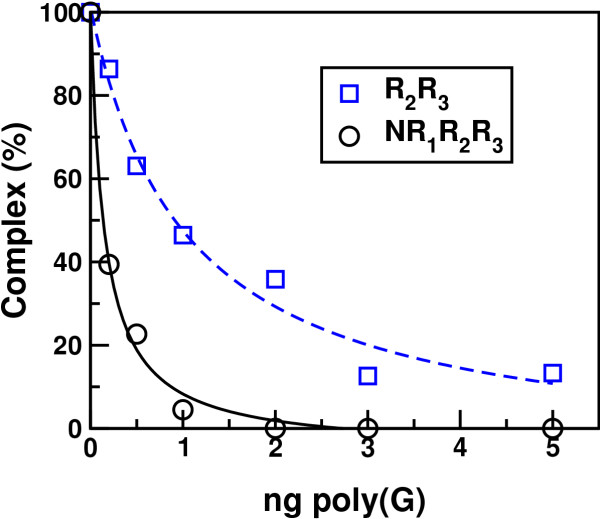
Titration of the poly(G) inhibition. NR_1_R_2_R_3 _(40 fmol) and R_2_R_3 _(20 fmol) were incubated separately with 20 fmol MRE-containing radiolabelled probe and increasing amounts of poly(G). The binding reactions were incubated for 15 minutes at 25°C and subsequently analyzed by EMSA and phosphorimaging. The intensities of the complex bands were quantified by phosphorimaging software and plotted as percentage of the complex band intensity when no homoribopolymers was added.

To better compare the sensitivity of the two proteins, we titrated the poly(G) inhibition (Fig. 3). The DNA-binding of NR_1_R_2_R_3 _was significantly more sensitive to poly(G) competition than R_2_R_3, _indicating that the N-terminus including the R_1 _repeat was important for the inhibitory effect.

**Figure 4:**
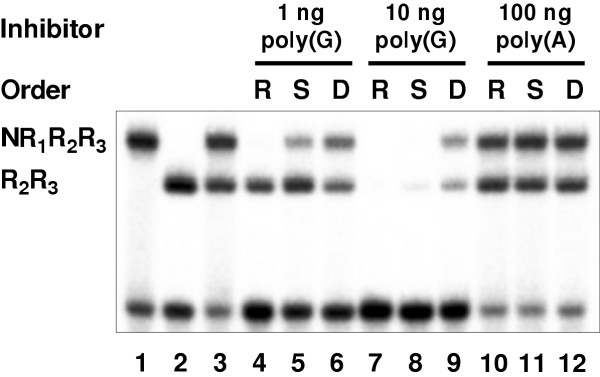
Order-of-addition analysis. A mixture of 40 fmol NR_1_R_2_R_3 _and 20 fmol R_2_R_3 _was incubated with 30 fmol MRE-containing DNA probe and 1 ng poly(G) (lane 4–6), 10 ng poly(G) (lane 7–9) and 100 ng poly(A) (lane 10–12). In the cases marked **R **(for "RNA first", lane 4, 7 and 10), the proteins were incubated with homoribopolymers for 15 minutes at 25°C before addition of DNA-probe and further incubation for 15 minutes at 25°C. The cases marked **S **(for ``simultaneous addition'', lane 5, 8 and 11) represents reactions where homoribopolymers and DNA probe were mixed before addition of protein and 15 minutes of incubation. Finally, the cases marked **D **(for ``DNA first'', lane 6, 9 and 12) show reactions where the protein were incubated with DNA first and then homoribopolymers were added. The reactions in lane 1–3 contained no homoribopolymers, NR_1_R_2_R_3 _protein alone in lane 1 and R_2_R_3 _protein alone in lane 2. The binding reactions were subsequently analyzed by EMSA and phosphorimaging.

Different plausible mechanisms for the observed poly(G) inhibition of c-Myb sequence-specific DNA-binding may be operating. Poly(G) might bind to c-Myb in direct competition with DNA. Or, the binding of poly(G) to c-Myb might be allosteric, inducing structural changes in c-Myb that reduces its DNA-affinity. A third explanation might be that poly(G) in a subtle way interacts with the DNA-probe and blocks its specific interaction with c-Myb. To clarify the mechanism of inhibition, we studied the importance of the order of addition of probe and homoribopolymers to the binding reaction (Fig. 4). Three situations were investigated, designated R, S and D in Fig. 4: (R) RNA was mixed with the proteins before addition of probe, (S) RNA and probe were mixed and added simultaneously to the proteins, (D) Probe (DNA) was mixed with protein before addition of RNA. The reasoning was that if the inhibition is allosteric of nature, the magnitude of inhibition should be independent of the order of addition. If probe-interference is the mechanism, the strongest inhibition should be observed with the simultaneous addition of RNA and probe. If a competitive binding of poly(G) to c-Myb is the case, the strongest inhibition should be expected with the addition of RNA to protein first and the weakest inhibition when the probe was added first. The last scenario was in fact what we observed, indicating that c-Myb binds to poly(G) in direct competition with DNA-binding (Fig. 4). No inhibition at all was observed when 100 ng of poly(A) was added. It is noteworthy that the relative sensitivity of the two Myb forms also changed as a function of the order of addition. Pre-incubation with RNA enhanced the difference between NR_1_R_2_R3 and R_2_R_3 _significantly, the first being fully inhibited while the latter seemed to be almost unaffected. In contrast, pre-incubation with DNA allowed the two forms to bind with similar efficiency. This supports the notion that the N-terminal region including the R_1 _repeat plays an important role in conferring RNA-sensitivity to the protein.

The homoribopolymer experiments reported above were performed with 15 minutes of incubation at 25°C. To exclude that the poly(G) effect was just a consequence of slower complex formation, we repeated the experiment with longer incubation times up to 90 minutes (results not shown). No change in the inhibition pattern was observed. This argues against the possibility that poly(G) only reduces the rate of c-Myb/DNA complex formation and indicates that the complex with RNA is highly stable.

**Figure 5:**
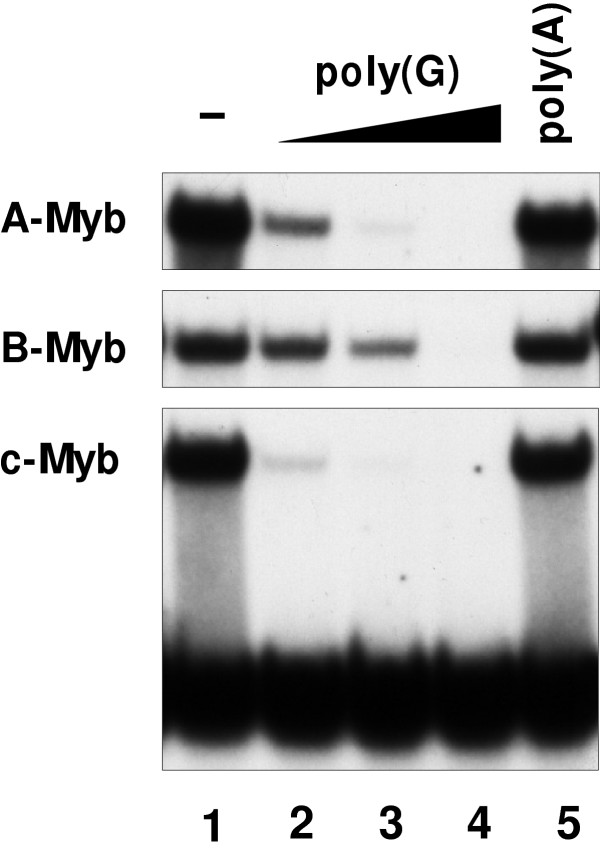
Comparison of A-, B- and c-Myb's poly(G) inhibition. A-, B- and c-Myb NR_1_R_2_R_3 _(40 fmol each) were incubated separately with 20 fmol radiolabelled MRE-containing DNA probe, 0.5 ng poly(G) (lane 2),1 ng poly(G) (lane 3), 5 ng poly(G) (lane 4) and 100 ng poly(A) (lane 5). Homoribopolymers and proteins were first incubated for 15 minutes at 25°C. Then the DNA-probe was added to the mixtures and subjected to a new incubation of the same time and temperature. The binding reactions were analyzed by EMSA and autoradiography. The extra incubation step without probe was added to the experimental setup because it enhanced the inhibitory effect (lanes marked R in Fig. 3).

We then asked whether the poly(G) inhibition was specific for c-Myb or if other Myb proteins exhibited the same properties. Purified NR_1_R_2_R_3 _forms of the vertebrate Myb relatives A- and B-Myb together with c-Myb were analyzed by EMSA in the presence of different concentrations of poly(G) and poly(A). As shown in Fig. 5, the DNA-binding of A- and B-Myb was clearly inhibited by poly(G), although the effect was somewhat weaker than for c-Myb. A-Myb behaved most similar to c-Myb, consistent with the close resemblance between these two transcription factors [46].

## Discussion

The DBD of c-Myb contains three tandemly arranged pseudo-repeats, R_1_, R_2 _and R_3_, among which R_2 _and R_3 _together are responsible for sequence-specific DNA-binding. The function of the first repeat, R_1_, has remained elusive in particular because R_1 _does not directly contact DNA. Its involvement in the process of oncogenic activation of Myb suggests a specific biological role of R_1_. In this report we have focused on the N-terminal region including the R_1 _repeat of c-Myb, trying to find a function beyond DNA-binding. A homoribopolymer inhibition assay revealed a strong preferential inhibition of sequence-specific DNA-binding by poly(G), suggesting that c-Myb can interact with RNA in a sequence-selective fashion. The most interesting observation was the finding that the RNA-interference function of c-Myb was highly dependent on the N-terminal region including R_1_. Upon exposure to RNA before DNA, the protein domain containing this region was severely inhibited by low concentrations of poly(G) while the protein domain lacking this region was almost unaffected. What the precise biological role of this novel RNA-interaction is, remains to be elucidated.

Central to our examination of the RNA-binding hypothesis is a homoribopolymer inhibition assay. Differential binding to homoribopolymers has been exploited as evidence for RNA-binding activity of several proteins, like the Ets-related transcription factor PU.1 [41], the neuronal KH domain containing protein Nova-1 [43], the chloroplast ribosomal protein CS1 [44], and the recently cloned RRM domain containing Ciona intestinalis protein RGC [45]. Whether homoribopolymer binding reflects a sequence-specific RNA binding or a more general RNA-binding, like in the case of polypyrimidine-tract or poly(A) binding proteins, requires further analysis in each case.

In addition to the data presented above, the SELEX (systematic evolution of ligands by exponential enrichment) technology was applied to search for more sequence-specific RNA patterns recognized by c-Myb. The SELEX procedure did produce specific patterns, confirming the proper behaviour of the experiment, but the selected RNAs did not seem to mimic the homoribopolymer effect in terms of inhibitory efficiency and content of G-bases (results not shown). This does not argue against RNA-binding per se, but indicates that c-Myb may not interact in a strictly sequence-specific fashion with RNA. Rather, we believe that the inhibitory RNA-effect seen in the homoribopolymer experiments reflects an R_1 _-dependent RNA-interaction where G-rich RNA interacts more avidly than other RNAs. Why G-rich species are so much more potent inhibitors is not obvious. G-rich RNA molecules have special folding capacities that could be recognized by c-Myb. This is illustrated by the fact that poly(G) has been reported to fold into several unique structures, including single, double and four-stranded helices [47-49]. To investigate the possibility that poly(dG) had a similar effect on c-Myb DNA binding as poly(G), we added DNA oligonucleotides containing deoxy-guanine stretches of varying length (1 to 5) to EMSA reactions. However, we were not able to correlate the presence of poly(dG) stretches to any inhibitory effects (results not shown). Neither did we observe any strong inhibition when poly(I*C) or poly(I) was added. It is quite probable therefore that unique structural properties of poly(G) are critical to the mechanism of inhibition.

The poly(G) inhibition was surprisingly strong. Due to the undefined length of the homoribopolymers, it was not evident how to precisely determine their molar concentrations. A fictitious poly(G)-length of 23 nucleotides, which is the length of the probe, gives an RNA-concentration of about 6 nM when 1 ng is added to the binding reactions. Consequently, a six-fold estimated molar excess of poly(G) compared to the probe concentration (1 nM) was sufficient to completely abolish sequence-specific DNA-binding (Fig. 3).

We also analyzed the effects of a series of mononucleotides (results not shown). Interestingly, specific nucleotide triphosphates did in fact inhibit DNA-binding of c-Myb with differences resembling the pattern observed with ribopolymers in the present work. GTP produced the most prominent effect among the nucleotides, while CTP or UTP had little or no inhibitory effect. However, since the inhibition was observed first in the mM concentration range, significantly higher than the amount of poly(G) producing the same level of inhibition, the GTP phenomenon seems to be only a weak reflection of the strong inhibition we see with poly(G). Still, it is intriguing that we observe the same specificity suggesting some type of specific interactions between guanosines and the DNA-binding domain of c-Myb.

The reported results represent evidence for an RNA-binding function of c-Myb. The dependence on the N-terminal region including R_1 _is not total, in the sense that without this region all RNA-interference disappears. Rather, the presence of this region seems to enhance the sensitivity to RNA-mediated inhibition several fold, making it possible to find conditions where a Myb DBD containing this region becomes fully inhibited while a minimal DBD remains more or less unaffected (Fig. 4). At higher concentrations of poly(G) RNA, however, both proteins become inhibited, suggesting that other parts of the DBD are involved too. An appealing hypothesis would be that the flexible second repeat were involved in interactions with both types of macromolecules, cooperating with R_1_ for RNA-binding and with R_3 _for DNA-binding. This would explain why this repeat appears to be a more flexible protein domain than the other repeats. It is noteworthy that a DNA-bound protein seems to be resistant to RNA-mediated inhibition and that an RNA-associated protein does not bind DNA even after prolonged incubation. We have previously shown that c-Myb R_2_R_3 _undergoes a conformational change upon binding to DNA [14,50,51]. It is possible that the DNA-induced conformation is resistant to RNA-interference and that RNA induces another conformation that is unable to bind DNA; in other words that the two nucleic acids lock the protein in two distinct conformations.

It could be argued that we have shown mainly experiments of the RNA-interference type, not directly demonstrating RNA-binding. We have, however, several lines of evidence indicating that RNA-interference occurs through direct binding of c-Myb DBD to RNA. c-Myb NR_1 _R_2 _R_3 _was observed to interact with RNA in a North-Western experiment where R_2_R_3 _did not, and c-Myb NR_1_R_2_R_3 _bound to RNA-linked beads (results not shown).

The identification of an increasing number of proteins capable of both DNA- and RNA-binding challenges the established picture of DNA-bound regulators with functions confined to promoter-activation, and suggests a broader function for some transcription factors [39]. The precise physiological role of the novel RNA-binding property of c-Myb remains to be elucidated. An interesting possibility to investigate is whether c-Myb plays a role beyond transcriptional activation in biological processes involving RNA, like splicing, capping, polyadenylation, nuclear export or transport of RNA. A role in one or several of these processes will fit the forthcoming model of a coupling between transcription and the post-transcriptional fate of mRNA [52,53].

## Conclusions

We have obtained evidence that c-Myb DNA-binding is preferentially inhibited by poly(G) RNA, indicative of a sequence-selective RNA binding function. The N-terminus of c-Myb, including the R_1 _repeat, was shown to contribute substantially to this RNA-sensitivity. This finding suggests a more specific function of the enigmatic first Myb repeat than having a stabilizing effect on DNA-binding only.

## Methods

### Homoribopolymers

All homoribopolymers were purchased from Sigma Aldrich. Purities were higher than 98% for all polymers. The length distributions of the homoribopolymers were determined by agarose gel electrophoresis and UV-shadowing. When compared to a dsDNA ladder, poly(A), poly(C), poly(G)and poly(U) migrated with a distribution corresponding to the following ranges: 200–1600 bp, 250–1200 bp, 100–700 bp and 250–700 bp, respectively (results not shown). RNase T1 was purchased from Ambion, Inc.

### Expression and purification of recombinant proteins

The following DBD subdomains were expressed in *E. coli *(strain BL21 (DE3) LysS) using the T7 system [54]: Human c-Myb residue 1–192 (NR_1_R_2_R_3_) and 89–192 (R_2_R_3_), human A-Myb 1–187 (NR_1_R_2_R_3_) and B-Myb 1–183 (NR_1_R_2_R_3_). Expression and purification were performed as previously described [46].

### Electrophoretic mobility shift assay

Sequence-specific DNA-binding was examined by electrophoretic mobility shift assay (EMSA) as previously described [55]. The binding reactions were performed in 20 mM Tris-HCl pH 8.0, 0.1 mM EDTA, 10% glycerol, 0.1 mM DTT, 0.005% Triton X-100 and 50 mM NaCl in a total volume of 20 μl. RNAguard (Amersham Biosciences) RNAse inhibitor (6–20 U) was added to each reaction to avoid degradation of the homoribopolymers.

The sequence of the MRE-containing DNA probe was from the *mim-1 *promoter: 5'-GCATTATAACGGTTTTTTAGCGC-3'. Double-stranded DNA oligonucleotides were ^32^P- labelled by T4 polynucleotide kinase according to the specifications of the manufacturer (Ready-To-Go T4 Polynucleotide kinase, Amersham Biosciences). Radiolabelled probe was purified on G-25 MicroSpin columns (Amersham Biosciences) or by polyacrylamide gel electrophoresis with subsequent gel extraction.

### Phosphorimaging

EMSA gels were transferred to 3 MM paper and dried for 90 minutes at 80°C in a vacuum gel drier. Radiolabelled bands were detected with Molecular Imaging Screen BI (Bio-Rad) and analyzed with a Bio-Rad GS-250 PhosphorImager. Quantitation of band intensities was performed with the Molecular Analyst 2.0.1 software (Bio-Rad).

## Authors' contributions

ON performed the majority of the experiments and participated in the writing of the manuscript. TØA was responsible for the experiments shown in Fig. 1B and 2. OSG designed the study and participated in the writing. All three authors read and approved the final manuscript.
